# Leveraging a gene signature associated with disulfidptosis identified by machine learning to forecast clinical outcomes, immunological heterogeneities, and potential therapeutic targets within lower-grade glioma

**DOI:** 10.3389/fimmu.2023.1294459

**Published:** 2023-12-15

**Authors:** Yao Zhou, Yudong Cao, Weidong Liu, Lei Wang, Yirui Kuang, Yi Zhou, Quan Chen, Zeyu Cheng, Haoxuan Huang, Wenlong Zhang, Xingjun Jiang, Binbin Wang, Caiping Ren

**Affiliations:** ^1^ National Health Commission (NHC) Key Laboratory of Carcinogenesis, Department of Neurosurgery, Xiangya Hospital, Central South University, Changsha, Hunan, China; ^2^ National Clinical Research Center for Geriatric Disorders, Xiangya Hospital, Central South University, Changsha, Hunan, China; ^3^ The Key Laboratory of Carcinogenesis and Cancer Invasion of the Chinese Ministry of Education, Cancer Research Institute, School of Basic Medical Science, Central South University, Changsha, Hunan, China; ^4^ Department of Neurosurgery, Xiangya Hospital, Central South University, Changsha, Hunan, China; ^5^ Department of Neurosurgery, The First Affiliated Hospital with Nanjing Medical University, Nanjing, China

**Keywords:** lower-grade glioma, disulfidptosis, prognostic signature, tumor microenvironment, ABI3

## Abstract

**Background:**

Disulfidptosis, a newly defined type of programmed cell death, has emerged as a significant regulatory process in the development and advancement of malignant tumors, such as lower-grade glioma (LGG). Nevertheless, the precise biological mechanisms behind disulfidptosis in LGG are yet to be revealed, considering the limited research conducted in this field.

**Methods:**

We obtained LGG data from the TCGA and CGGA databases and performed comprehensive weighted co-expression network analysis, single-sample gene set enrichment analysis, and transcriptome differential expression analyses. We discovered nine genes associated with disulfidptosis by employing machine learning methods like Cox regression, LASSO regression, and SVM-RFE. These were later used to build a predictive model for patients with LGG. To confirm the expression level, functional role, and impact on disulfidptosis of ABI3, the pivotal gene of the model, validation experiments were carried out *in vitro*.

**Results:**

The developed prognostic model successfully categorized LGG patients into two distinct risk groups: high and low. There was a noticeable difference in the time the groups survived, which was statistically significant. The model’s predictive accuracy was substantiated through two independent external validation cohorts. Additional evaluations of the immune microenvironment and the potential for immunotherapy indicated that this risk classification could function as a practical roadmap for LGG treatment using immune-based therapies. Cellular experiments demonstrated that suppressing the crucial ABI3 gene in the predictive model significantly reduced the migratory and invasive abilities of both SHG44 and U251 cell lines while also triggering cytoskeletal retraction and increased cell pseudopodia.

**Conclusion:**

The research suggests that the prognostic pattern relying on genes linked to disulfidptosis can provide valuable insights into the clinical outcomes, tumor characteristics, and immune alterations in patients with LGG. This could pave the way for early interventions and suggests that ABI3 might be a potential therapeutic target for disulfidptosis.

## 1 Introduction

Gliomas are identified as a cancerous condition that starts in the central nervous system, known for their rapid growth and invasive tendencies (1), leading to a decline in patients’ quality of life and lower chances of survival (2). As per the World Health Organization (WHO) classification, gliomas are categorized into four grades. Among these, grade II and III gliomas are referred to as lower-grade glioma (LGG) (3). Besides long-term exposure to ionizing radiation, the factors contributing to the risk of LGG are not fully understood (4). Based on a study (5), patients with grade 2 LGG experienced a median overall survival (OS) of 78.1 months, whereas those with grade 3 had a median OS of 37.6 months. Despite significant progress in the advancement of groundbreaking cancer therapies, the outlook for individuals with LGG remains grim. Immunotherapy is increasingly recognized as a promising treatment modality across a diverse spectrum of tumors. Therefore, the pressing need for the advancement and authentication of innovative predictive indicators to better predict medical results and guide immunotherapy approaches in individuals with LGG remains.

Apoptosis acts as a natural process controlling biological growth and preserving internal environmental balance. The strategic targeting of cell death-associated pathways to eradicate cancer cells constitutes a pivotal focus in oncological therapeutics (6). Disulfidptosis, a novel form of cell death, was recently discovered in a study at MD Anderson Cancer Center (7). This novel form of cellular demise differs from the recognized methods of demise. Once disulfidptosis occurs, it cannot be prevented by using conventional cell death inhibitors or by knocking down pivotal cell death-related regulatory genes, so it is completely independent of several other known modes of cell death. It was found that disulfidptosis is triggered by the rapid depletion of NADPH in SLC7A11-overexpressing cells during glucose deprivation, which leads to disulfide bond stress, amplifying the quantity of disulfide bonds within the actin cytoskeleton. Consequently, actin filaments contract, resulting in cytoskeletal structural disruption and precipitous cellular death. SLC7A11 is a member of the solute carrier family and is part of the cystine/glutamate reverse transporter proteins. These proteins primarily participate in the transportation of amino acids through the cell membrane, serving as a crucial pathway that tumor cells rely on for survival. Specifically, the conversion of cystine to cysteine by SLC7A11 heavily relies on NADPH produced through the glucose-pentose phosphate pathway (8). The disulfidptosis mechanism stands out from other cellular death pathways because of its distinct association with the actin cytoskeleton, an essential cellular structure for maintaining cell shape and survival. The actin cytoskeleton comprises actin filaments, pivotal in defining the cell’s structural integrity and overall form (7, 9). Furthermore, it was noted that the application of glucose transporter inhibitors resulted in a significant decrease in cellular glucose uptake in cancer cells that overexpress SLC7A11. This reduction subsequently resulted in NADPH depletion, actin cytoskeleton cross-linking, and the initiation of disulfidptosis.

Furthermore, it was noted that the application of glucose transporter inhibitors resulted in a significant decrease in cellular glucose uptake in cancer cells that overexpress SLC7A11. This reduction subsequently resulted in NADPH depletion, actin cytoskeleton cross-linking, and the initiation of disulfidptosis (10). Likewise, disulfidptosis has been shown to have the ability to impact the infiltration of immune cells (11). Due to the early phase of investigation into disulfidptosis, its impact on the advancement of illnesses in individuals with LGG is still uncertain. Although ongoing studies have only identified a limited number of genes associated with disulfidptosis (DAGs), the medical community has shown considerable interest in the concept of disulfidptosis since its beginning, especially in the field of tumor therapy (12). Therefore, exploring the possible connection between DAGs and the cause of LGG has significant value in the advancement of focused treatment strategies for LGG (8).

In this inquiry, we obtained publicly accessible LGG information from The Cancer Genome Atlas (TCGA) and Chinese Glioma Genome Atlas (CGGA) repositories. By utilizing various bioinformatics techniques, such as Weighted Gene Co-expression Network Analysis (WGCNA), single-sample genome enrichment analysis (ssGSEA), and machine learning methods, we effectively developed an innovative risk model using nine DAGs. Additional evaluation was performed to assess the predictive usefulness of this risk model and explore its connections with immune response against tumors and the microenvironment of the tumor. After extensive experimentation, we have definitively determined that the ABI3 protein is intricately connected to vital mechanisms in glioma cells, encompassing movement, infiltration, and the initiation of epithelial-mesenchymal transition. Moreover, our research indicates that ABI3 could be a potential target for promoting disulfidptosis therapeutically.

## 2 Materials and methods

### 2.1 Data acquisition and processing for transcriptomes

We extracted the RNA expression profiles, gene mutations, and relevant clinical data for LGG from the databases of TCGA. The TCGA datasets were used as the training set to construct the model. To validate externally, RNA transcriptomics data from two glioma cohorts were obtained from the CGGA databases (13, 14). The training dataset aided in the creation of a predictive model, whereas the external validation dataset evaluated the model’s strength and accuracy in prediction. For analysis purposes, the data were presented in FPKM format, which stands for Fragments Per Kilobase of transcript per Million mapped reads, and then transformed using a logarithmic function. Furthermore, the RNA-seq information of healthy brain tissue was obtained from the GTEx database (https://commonfund.nih.gov/GTEx). The ‘sva’ package in R corrected the batch effect on the TCGA, GTEx, and CGGA datasets.

### 2.2 Analysis of gene set enrichment for individual samples using ssGSEA

To calculate the disulfidptosis scores for individual LGG samples, the Gene Set Enrichment Analysis (GSEA) utilized the ‘GSVA’ and ‘GSEABase’ packages in R. The gene set associated with disulfidptosis was carefully selected based on an extensive review of relevant literature (7). **Supplementary Table 1** contains the exhaustive compilation of these genes.

### 2.3 Evaluation of weighted co-expression networks

The TCGA-LGG dataset was used to construct gene co-expression networks through the execution of the WGCNA using the ‘WGCNA’ package in R. By examining the connectivity within each set of genes and its correlation with phenotypic traits, WGCNA allows for the identification of highly co-expressed gene modules as well as potential biomarker genes or therapeutic targets. In particular, the WGCNA method was utilized to detect gene modules that are associated with disulfidptosis scores in LGG patients and to describe these genes within the identified modules. Additionally, in order to ensure WGCNA’s rationality, we performed a hierarchical cluster analysis to exclude outliers.

### 2.4 Genes intersecting through differential expression analysis and Venny analysis

Differential expression analysis was conducted using control samples of healthy brain tissue obtained from the GTEx database. To identify differentially expressed genes (DEGs) (15), the R package ‘limma’ was utilized. After applying the Benjamini-Hochberg method for multiple hypothesis testing adjustments, DEGs were chosen by considering an absolute log2 fold change (|log2FC|) greater than 1.585 and an adjusted p-value lower than 0.05, ensuring a false discovery rate of less than 0.05. The ‘ggplot2’ R package was used to create a volcano map for the visualization of DEGs. To facilitate further analyses, a Venn diagram was utilized to determine the overlap between important module hub genes obtained from WGCNA and the DEGs.

### 2.5 Development and validation of an ideal machine learning-based prognostic risk model for DAGs

To begin with, a univariate Cox regression analysis was conducted to identify genes with prognostic importance by utilizing the hub genes associated with disulfideptosis that were differentially expressed. Furthermore, a comprehensive examination of two machine learning algorithms was employed to screen these predictive genes. The LASSO algorithm, which utilized penalty parameter tuning through 10-fold cross-validation, and the SVM-RFE algorithm, which selected the optimal variables based on the minimum 10-fold cross-validation error value, were the two algorithms used. A prognostic signature was developed by performing multivariate Cox survival analysis on the extracted and selected overlapping genes using the LASSO and SVM-RFE algorithms. To calculate prognostic signature risk scores, the following formula was utilized: $Risk\;scores=\sum_{i=1}^{n}\left(Coef_{i}*Exp_{i}\right)$ , Where ‘i’ denoted the serial number of gene, ‘Coef’ represented the coefficient value, and ‘Exp’ represented the expression value of gene.

It is crucial that the prognostic signature is validated by another independent cohort. As a result, the strength of our predictive pattern was confirmed by validating it on two separate glioma datasets (mRNAseq_325 and mRNAseq_693) obtained from the CGGA database. The TCGA- LGG cohort and two CGGA cohorts were divided into high-risk and low-risk groups based on the median risk score obtained from the TCGA cohort. Following assessments involved the utilization of Kaplan-Meier survival analysis and Receiver Operating Characteristic (ROC) curve analysis to evaluate the variations in prognosis between the high-risk and low-risk subcategories, as well as the precision of the risk model’s predictions.

### 2.6 Development and assessment of a prognostic risk scoring nomogram utilizing factors related to prognosis

The Cox model was used to assess independent predictors of OS. The variables that showed a strong correlation with prognosis in the univariate Cox analysis were later included in the multivariate Cox analysis. Furthermore, the Cox proportional hazards models were used to calculate hazard ratios (HRs) and their corresponding 95% confidence intervals (CIs).

To predict the chances of 3-, 5-, and 7-year OS in patients with LGG, a nomogram was developed. The ‘rms’ and ‘regplot’ packages in R were used to create this model, which combines the recognized independent prognostic clinical factors and the risk score signature. To evaluate the reliability and precision of the developed nomogram, calibration curves and decision curve analysis (DCA) were utilized. Dynamic nomograms were created using the “DynNom” package, and an interactive web-based application was created using the Shiny platform (https://yudong-cao.shinyapps.io/dynamic-Nomo/) (16).

### 2.7 Gene set variation analysis

The R package ‘GSVA’ was used to perform gene set variation analysis with the ‘c2.cp.kegg.v7.5.1.symbols.gmt’ dataset obtained from the Molecular Signatures Database. The R package ‘heatmap’ was used to generate a heat map for visualizing the enrichment results. Statistical significance was determined at an adjusted p-value of less than 0.05 using the “limma” R package.

### 2.8 Evaluation of the prognostic model’s relevance to tumor immunity and immunotherapeutic response

To determine the makeup of 22 different types of immune cells in humans, the CIBERSORT algorithm was employed by analyzing gene expression profiles (17–19). For successful deconvolution, a P-value of less than 0.05 was used to determine the statistical significance after executing 1,000 permutations for each sample.

The ESTIMATE algorithm is specifically created to measure the amount of infiltrating immune and stromal cells present in tumor tissue, while also providing an estimation of tumor purity based on gene expression data (20). Using ssGSEA, ESTIMATE calculates scores for immune, stromal, and tumor purity. The application of this tool was used to assess the tumor purity, immune score, and stromal score of every LGG sample. Furthermore, the ssGSEA was employed to calculate numerical values for every individual stage in a seven-stage cancer immunity cycle, which acts as a structure for assessing anti-cancer immune responses (21).

The TIDE algorithm is an online tool created to measure response metrics of immunotherapy, acting as a predictive tool to evaluate the efficacy of immune checkpoint inhibitors, and performing thorough analyses on various tumor expression profiles. By employing this tool, we computed several immune scores, such as immune malfunction, immune segregation, cancer-related fibroblast (CAF), and the comprehensive TIDE score. These scores were derived from standardized expression profiles obtained from the TCGA repository (22). To assess disparities in TIDE scores between subgroups at high and low risk, a Wilcoxon test was conducted.

### 2.9 The mutational landscape and drug sensitivity

The gene mutation patterns of patients with LGG, acquired from the TCGA database, were produced utilizing the ‘maftools’ application. Afterwards, the comprehensive gene mutation records were combined with the risk assessment. Additionally, we utilized the R software ‘oncoPredict’ to calculate the IC50 values, representing the concentrations at which the inhibitory effect of commonly used chemotherapeutic drugs is reduced by half. The assessment enabled the examination of the correlation between the risk rating and the responsiveness to medication. Wilcoxon signed-rank tests were conducted to compare the IC50 values between the two risk groups.

### 2.10 Analysis of single-cell RNA sequences

The Tumor Immune Single-Cell Hub (TISCH) was employed to examine scRNA-seq data of the GSE148842 dataset. TISCH functions as a dedicated repository for scRNA-seq, specifically targeting the TME [15]. By providing comprehensive annotations of cell types at the individual cell level, it enables the exploration of the TME in different types of cancers (23).

### 2.11 Cell culture

The glioma cell lines SHG44 and U251 were utilized in the experiments and were cultured in DMEM (Dulbecco’s Modified Eagle Medium) with the addition of 10% fetal bovine serum. The cells were kept in a typical sterile CO2 incubator at a temperature of 37°C, with a CO2 concentration of 5%. Every day, the cells were rinsed thrice using PBS (Phosphate-Buffered Saline), and the culture medium was substituted. When the cell confluency reached 80%-95%, subculturing was carried out with a split ratio of 1:2.

### 2.12 Tissue microarray and immunohistochemistry

The Ethics Committee of Xiangya Hospital, Central South University granted ethical approval for this study (Ethical Approval Code 202309185). Informed consent was obtained from all patients who participated. All participants were included in the study. During the time frame from January 2021 to August 2022, the Department of Neurosurgery at Xiangya Hospital, Central South University, obtained a total of 70 glioma samples and 12 normal tissues from the surrounding area. Following previous protocols, the specimens were processed into tissue microarrays to investigate the expression of ABI3 (24).

To remove paraffin and rehydrate, tissue microarrays embedded in paraffin were baked at a temperature of 65°C for one hour. The endogenous peroxidase activity was eliminated by incubating in methanol with 0.3% hydrogen peroxide for 30 minutes. Afterwards, the sections were obstructed for 30 minutes using a solution of 2% BSA in PBS. The anti-ABI3 antibody (diluted 1 250; SANTA, USA) was incubated overnight at 4°C.The MaxVision HRP-polymer IHC Kit Detection System (peroxidase/DAB, rabbit/mouse) from MaxVision, Fuzhou, China, was utilized to visualize immunocomplexes, following the guidelines provided by the manufacturer. Hematoxylin (Beyotime Biotechnology) was employed for nuclear counterstaining. Evaluation of staining was conducted using an optical microscope with a magnification of 200×.We conducted a semi-quantitative evaluation of ABI3 protein expression using methodologies that were previously established in our research (25).

### 2.13 Cell transfections and real-time quantitative PCR

The cells were placed in a 6-well dish with a seeding density of 2.5×105 cells per well. The cells were transfected with 5μl of ABI3 SiRNA (The siRNAs targeting the ABI3 gene were synthesized by RiboBo Corporation (Guangzhou, China)) using Lipofectamine 3000 Reagent (Invitrogen, Carlsbad, CA, USA) following the manufacturer’s instructions when they reached a confluency of 60% to 80%.

The Trizol lysis method was used to extract total RNA from SHG44 and U251 cells that were treated. The Thermo Scientific RevertAid First Strand cDNA Synthesis Kit (Thermo Scientific, Waltham, MA) was utilized for the synthesis of cDNA. RNA levels of ABI3 andβ-Actin were analyzed by the 2−ΔΔCt method. The primers were created by The Beijing Genomics Institute (BGI) and the sequences were designed as follows: for ABI3, the forward primer was 5’-CAGGTGGAAGCCCGTGTAAG-3’ and the reverse primer was 5’-AGTGGCTAAGGTGCCGATCTC-3’. The forward primer for β-Actin was 5’-CATGTACGTTGCTATCCAGGC-3’ and the reverse primer was 5’-CTCCTTAATGTCACGCACGAT-3’.

### 2.14 Western blotting assay

The Western blotting assay was performed in accordance with the instructions provided by the manufacturer. Western blotting utilized antibodies targeting ABI3 (sc-376982, SANTA), β-Actin (20536-1-AP, Proteintech), vimentin (10366-1-AP, Proteintech), and ZO-1 (21773-1-AP, Proteintech).

### 2.15 Wound-healing assessment

Prior to conducting the cell migration assay, U251 and SHG44 cells underwent interference treatment. When the cell density reached 80%-90%, a 100 µl pipette tip was utilized to generate four intersecting lines in every well, producing a crisscross pattern that resembled a grid. After the line was created, the culture medium was substituted with either a serum-free or low-serum medium. Subsequently, 8-10 positions were selected and recorded under an imaging microscope. Thereafter, at 24-hour intervals, the same positions were imaged using the imaging microscope. The experiment involved capturing multiple time points (2, 3). The distances of migration were measured using suitable software for statistical analysis based on the captured images.

### 2.16 Invasion and migration experiments with Transwell

Migration and invasion experiments were conducted in a 24-well plate using 8.0 μm pore inserts (Millipore, Bedford, MA, USA).2 million cells were placed in the upper chamber of the Transwell insert for the migration experiment. To perform the invasion test, Matrigel-coated filters (BD Biosciences, Franklin Lakes, NJ, USA) were utilized. For migration and invasion assays, cells were incubated for 24 and 48 hours, respectively. After incubation, the cells that migrated and invaded were fixed and stained using 0.1% (w/v) crystal violet. Using high-power microscopy, the number of cells that were chosen randomly, migrated, and invaded were counted.

### 2.17 Cell viability and colony-forming assays

1000 cells were cultured in 96-well plates, each sample was repeated 5 times, and the number of cells was calculated daily using the CCK-8 assay (A311-02, China Nanjing Novizan Biotechnology Co., Ltd.) for 5 days. For colony formation assay, cells were cultured in 6-well plates at a density of 500 cells per well for 2 weeks. The medium was changed every 3 days. After 2 weeks, the medium was removed and the cell colonies were stained with crystal violet (0.1%, 20% methanol) for 10 minutes.

### 2.18 Cell immunofluorescence staining

The U251 and SHG44 glioma cell lines were taken out of the culture medium and rinsed twice with PBS. Afterwards, they were subjected to 3.7% formaldehyde treatment for a duration of 20 minutes, followed by 2-4 washes using PBS containing 0.1% Triton X-100. Each wash lasted for 5 minutes. The Actin-Tracker Red (C2205s, Beyotime) was thinned in PBS with 1-5% BSA and 0.1% Triton X-100 at a proportion of 1: 40-200. Following that, the cells were placed in a dark room at room temperature and incubated for 60 minutes. DAPI (Sigma, USA) was used to stain the cell nuclei. Observations were made using a laser confocal microscope.

### 2.19 Statistical analysis

GraphPad Prism software (version 9.0) was utilized to perform statistical analysis on the experimental data. The data were obtained from three separate experiments and are presented as the mean plus or minus the standard deviation (SD). Student’s t-tests were conducted for intergroup comparisons, with significance levels indicated as *P< 0.05, **P< 0.01, and ***P< 0.001.

## 3 Results

### 3.1 Weighted co-expression network analysis

**Figure 1:**
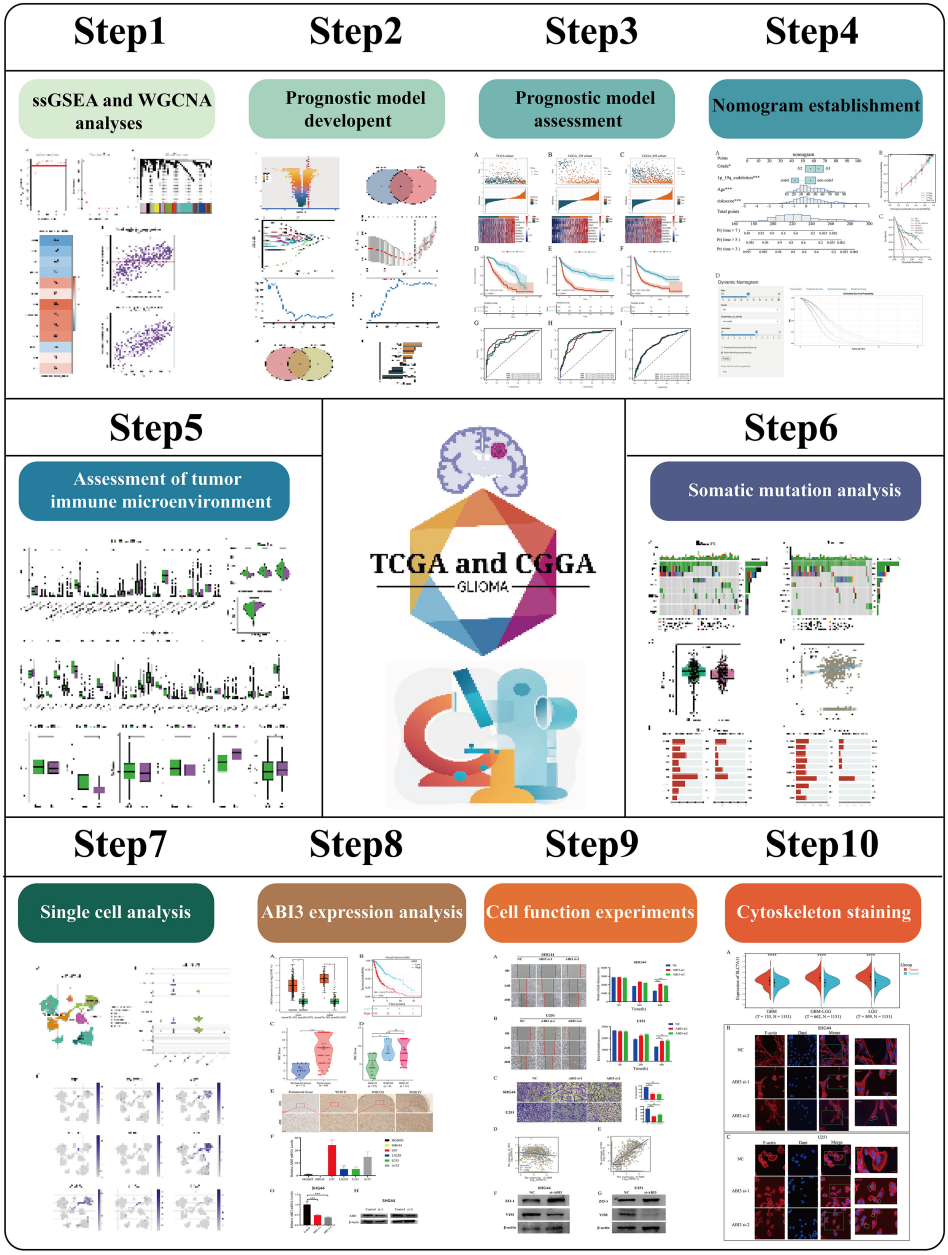
The flow diagram of this project.

**Figure 2:**
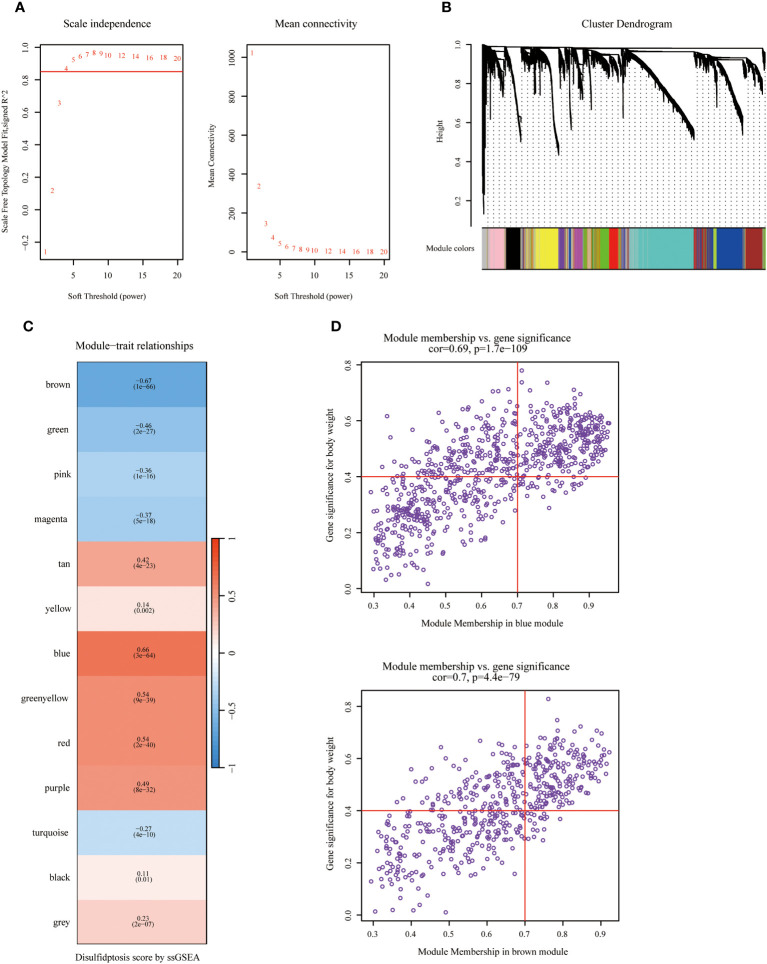
Identifying hub genes related to disulfidptosis phenotype via Weighted Co-Expression Network Analysis (WGCNA). **(A)** Network topology across varied soft thresholding powers. The figure numerically annotates the specific soft thresholding powers applied. An approximate scale-free network topology was observed at a soft thresholding power of 4. **(B)** Dendrogram of gene clusters based on topological overlap dissimilarity. Associated module colors are denoted in the color column. Each colored column signifies a module comprising a cluster of co-expressed genes. Thirteen distinct modules were identified. **(C)** Relationship between module characterization genes and disulfidptosis scoring phenotypes. **(D)** Association scatterplot between Module Membership and Gene significance in the blue and brown modules.

**Figure 1** displayed the schematic diagram of the research process. WGCNA was applied to systematically identify gene modules that exhibit covariance with disulfidptosis. As evidenced by **Figure 2A**, the adoption of a soft-thresholding value of 4 results in data that more closely adhere to a power-law distribution, while also stabilizing average network connectivity. This renders the dataset amenable to subsequent analyses. As depicted in **Figure 2B**, twelve distinct non-gray modules were generated through the amalgamation of modules exhibiting similarity coefficients below 0.15, with a minimum module size established at 50 genes and a deepSplit parameter set to 2. As discerned from **Figures 2C, D**, the brown and blue modules exhibit the strongest correlation with disulfidptosis, with correlation coefficients of -0.67 and 0.66, respectively. **Figure 2D** delineates the statistical significance of gene constituents within the brown and blue modules in relation to their correlation with disulfidptosis scores. Furthermore, WGCNA was also employed to pinpoint hub genes within brown and blue modules, resulting in the identification of 181 key hub genes.

### 3.2 Development and verification of a predictive signature utilizing DAGs

**Figure 3:**
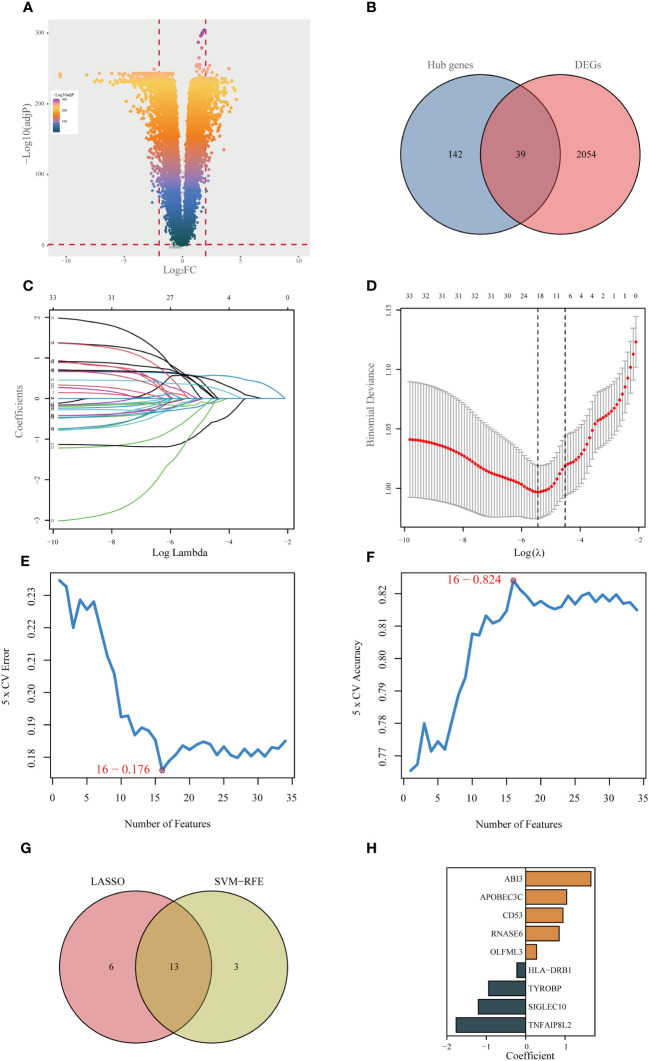
Identifying the best predictive model through machine learning. **(A)** Differential expression analysis between lower-grade glioma (LGG) and normal tissue (GTEx database). **(B)** Acquisition of 39 essential disulfidptosis-associated genes (DAGs) after intersecting hub genes obtained in WGCNA and differentially expressed genes (DEGs). **(C, D)** LASSO algorithm identifies 19 important DAGs. **(E, F)** The SVM-RFE algorithm selected 16 authoritative DAGs. **(G)** The intersection of genes obtained in LASSO and SVM-RFE algorithms. **(H)** Construction of 9-DAG signature via Cox regression analysis.

Initially, an integrated analysis of TCGA and GTEx databases was performed, yielding 2093 DEGs specific to LGG (**Figures 3A**, **Supplementary Table 2**). A Venn diagram was employed to intersect the DEGs with the hub genes identified via WGCNA, identifying 39 vital regulatory genes (**Figures 3B**, **Supplementary Table 3**). Following the execution of univariate Cox regression analysis, a subset of 34 genes was retained, each demonstrating a significant correlation with prognosis (**Supplementary Table 4**). Subsequently, upon isolating 34 prognostic genes, the LASSO method was applied to refine this list to 19 essential genes (as shown in **Figures 3C, D**), and the SVM-RFE algorithm was employed to narrow down the list to 16 essential genes (**Figures 3E, F**). Upon intersecting the critical genes identified through both LASSO and SVM-RFE algorithms, a set of 13 potentially important genes emerged (**Figure 3G**). These genes were subsequently subjected to multivariate Cox regression analysis for further refinement and the construction of 9-gene prognostic signature using the following equation: Risk Score= 1.656 × expABI3 + 1.041 × exp APOBEC3C + 0.949 × expCD53 + 0.851 × expRNASE6 + 0.276 × expOLFML3 - 0.222 × expHLA-DRB1 - 0.942 × expTYROBP - 1.201 × expSIGLEC10 - 1.763 × expTNFAIP8L2 (**Figures 3H**).

**Figure 4:**
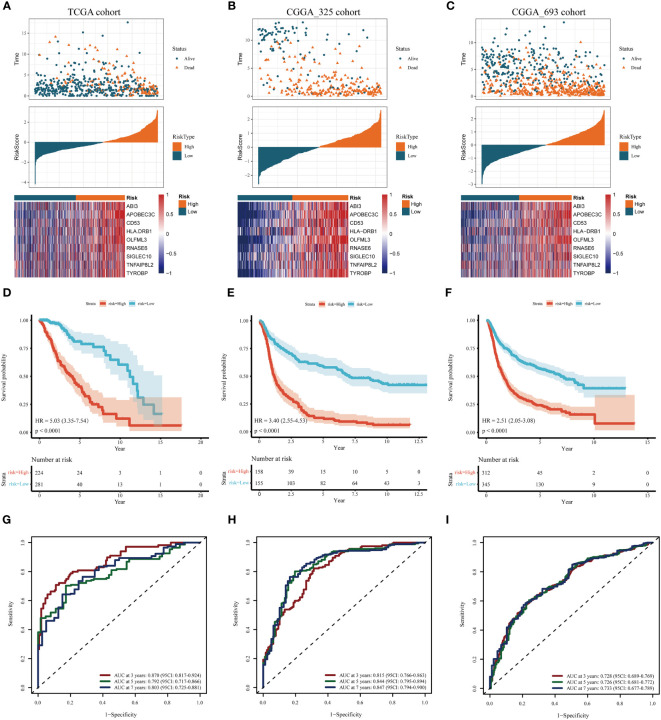
Assessment and validation of the prognostic significance of risk score. **(A–C)** Distribution of risk scores, the status of patient survival, and the expression patterns of the nine DAGs included inside the signature in the TCGA training set and the two CGGA validation sets, respectively. **(D–F)** The Kaplan-Meier (KM) survival analysis in the TCGA training and the two CGGA validation sets, respectively. **(G–I)** The receiver operating characteristic (ROC) curve analysis in the TCGA training and the two CGGA validation cohorts, respectively.

In the training cohort, samples were segregated into low-risk and high-risk categories, as illustrated in **Figure 4A**. The incidence of mortality was significantly lower in the low-risk set than in the high-risk set (**Figure 4A**).

The heatmap delineates that the expression levels of the nine model genes were markedly elevated in the high-risk subgroup compared to the low-risk subgroup within the TCGA cohort (**Figure 4A**). These findings were corroborated through analysis of two independent databases, namely the CGGA_325 and CGGA_693 datasets (**Figures 4B, C**).

As depicted in **Figures 4D-F**, the prognosis for patients categorized into the high-risk group was significantly poorer than those in the low-risk group across both the TCGA training cohort and the two CGGA validation cohorts (*P<* 0.001). This evidence substantiates that the disulfidptosis-related prognostic model exhibits high accuracy in forecasting patient outcomes across both the TCGA and CGGA cohorts. ROC curve analysis was conducted in the training set and the two test sets to further evaluate the predictive accuracy of this signature. The areas under the curve for the test group at 3, 5, and 7 years were observed to be 0.87, 0.79, and 0.80, respectively (**Figure 4G**). The Area Under the Curve (AUC) exceeded 0.72 for both the two CGGA test sets, indicating robust prognostic accuracy (**Figures 4H, I**).

### 3.3 Clinicopathological evaluation of the risk model and analysis of drug sensitivity

**Figure 5:**
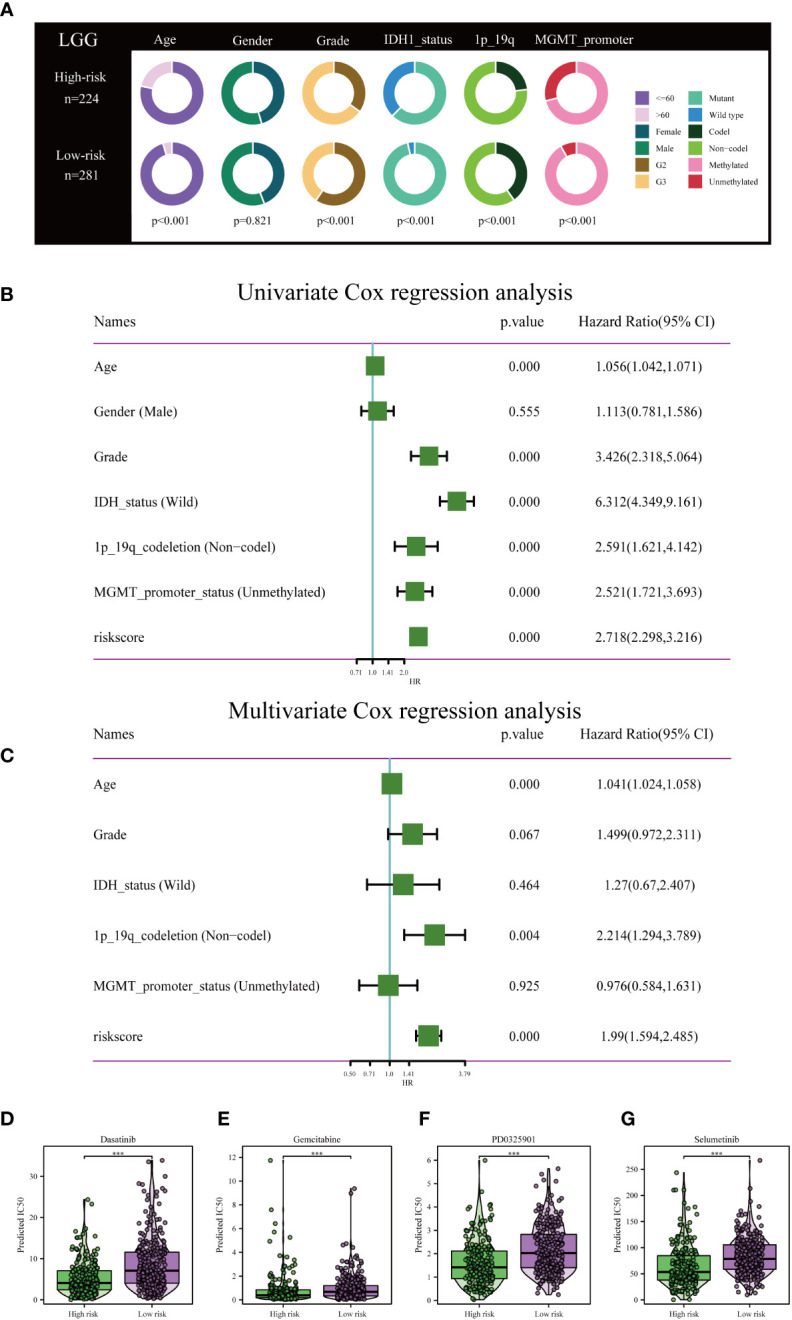
Independent prognostic assessment of risk scores and clinical parameters, and drug susceptibility prediction. **(A)** Chi-Square test depicting clinical and pathological characteristics across high-risk and low-risk subgroups within the TCGA cohort. The graphical representation employs circles to delineate the statistical test outcomes. **(B)** Prognostic factors for patients with LGG in the TCGA cohort were identified via Univariate Cox regression analysis. **(C)** Independent prognostic factors were further determined by multifactorial Cox regression analysis. Drug sensitivity analysis of Dasatinib **(D)**, Gemcitabine **(E)**, PD0325901 **(F)**, and Selumetinib **(G)** in patients with low and high risk scores. ***P< 0.001.

Clinical circle plots were constructed to elucidate disparities in clinicopathological features between the low-risk and high-risk groups within the context of LGG, and the analysis revealed an asymmetric distribution across all examined features with the exception of gender (**Figure 5A**). Patients presenting with higher pathological grade, advanced age, wild-type IDH1, absence of 1p/19q co-deletion, and un-methylated MGMT promoter were primarily allocated to the high-risk category (**Figures 5A**).

To rigorously validate the predictive efficacy of the disulfidptosis-related risk model in conjunction with other clinical features, both univariate and multivariate Cox regression analyses were conducted within the TCGA training cohort. Univariate Cox regression analysis revealed that both the risk scores and specific clinical parameters—namely age, tumor grade, IDH1 mutation status, 1p/19q co-deletion status, and MGMT promoter methylation status—exhibited significant associations with the prognosis of LGG, as depicted in **Figure 5B**. Multivariate Cox regression analysis substantiated that the risk score persisted as an independent prognostic factor (HR = 1.99, 95% CI 1.594-2.485, *P<* 0.001), as illustrated in **Figure 5C**. Conventional clinical attributes, such as age (HR = 1.041, 95% CI 1.024-1.058, *P<* 0.001) and 1p/19q co-deletion status (HR = 2.214, 95% CI 1.294-3.789, P = 0.004), were also verified as independent determinants of OS (**Figure 5C**).

Further investigation was conducted to elucidate the disparities in drug resistance among the two risk groups, as depicted in **Figures 5D-G**. According to our analysis, Gemcitabine, Dasatinib, Selumetinib, and PD0325901 have shown promise as potential treatment options for individuals classified as high-risk.

### 3.4 Construction of a prognostic nomogram

**Figure 6:**
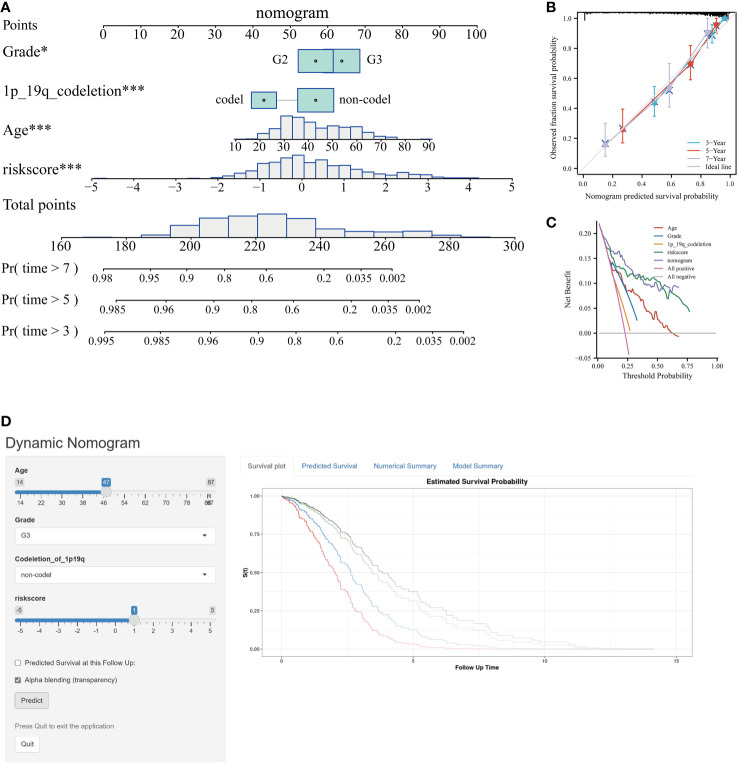
Developing and validating of nomogram based on risk scores. **(A)** Predicting 3-, 5-, and 7-year survival of LGG patients in the TCGA database using conventional nomogram. **(B)** The calibration curves for predicting 3-, 5- and 7-year overall survival (OS). **(C)** Decision curve analysis (DCA) for the nomogram in 3‐year OS. **(D)** Printscreen of the intuitive interface of the online dynamic nomogram for OS. *P < 0.05, ***P < 0.001.

Incorporating the risk score along with all independent prognostic clinical variables, a nomogram was constructed to quantify better the risk associated with LGG patients (**Figure 6A**). A nomogram was employed to enhance the accuracy of patient risk determination, thereby informing subsequent treatment strategies. Furthermore, the clinical prediction models were assessed and optimized through decision and calibration curve analyses, offering a comprehensive evaluation of their value in clinical decision-making. The findings demonstrated that the performance of the nomogram surpassed other clinical markers, indicating its robust capability in prognostic prediction (**Figures 6B, C**). Consequently, it holds the potential for utilization as a clinical decision-support instrument. Besides, the nomogram was further developed into an online, interactive tool to streamline the risk assessment process and facilitate user engagement and clinical decision-making (https://yudong-cao.shinyapps.io/dynamic-Nomo/) (**Figure 6D**). The web server is designed to generate estimated survival rates and Kaplan-Meier curves upon entry of the relevant covariates.

### 3.5 Analyses of pathway enrichment in cohorts with high- and low-risk

**Figure 7:**
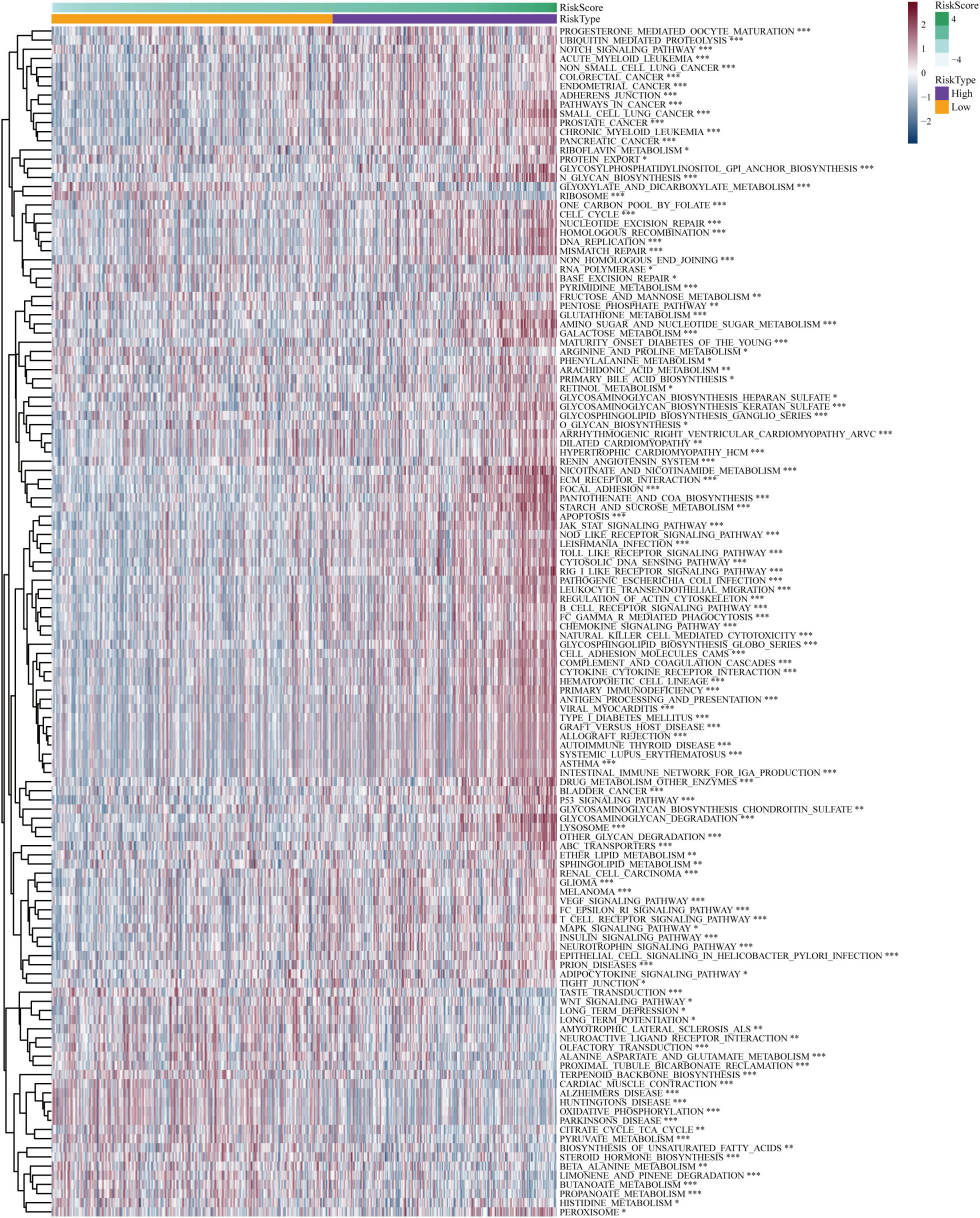
Differential Gene Set Variant Analysis (GSVA) enrichment items for high-risk and low-risk subgroups. *P< 0.05, **P< 0.01, ***P< 0.001.

The “c2.cp.kegg.v7.5.1.symbols” file was acquired from the Molecular Signature Database, and subsequent GSVA analysis was conducted utilizing this gene set to gain initial insights into the potential mechanisms underlying the impact of the risk score on prognosis in LGG patients. The GSVA results indicated that the high-risk group exhibited significant enrichment in biological processes encompassing signaling pathways related to DNA replication and damage repair, immune response, apoptosis, cell signaling, cell-cell interactions, and cell-matrix interactions, such as notch signal pathway, adherens junction, pathways in cancer, DNA replication, non-homologous end joining, focal adhesions, jak stat signaling pathway, p53 signaling pathway, MARK signaling pathway, and regulation of the actin cytoskeleton (**Figure 7**). Biochemical and metabolic pathways were predominantly enriched in the low-risk group. The aforementioned findings reaffirmed that the disulfidptosis-related genes utilized for risk score calculation play crucial roles in malignant progression, metabolism, DNA damage repair, immune responses, cell communication, and cytoskeleton regulation of glioma.

### 3.6 The disulfidptosis-based signature exhibits a correlation with heterogeneous TME and differential responses to immunotherapeutic interventions

**Figure 8:**
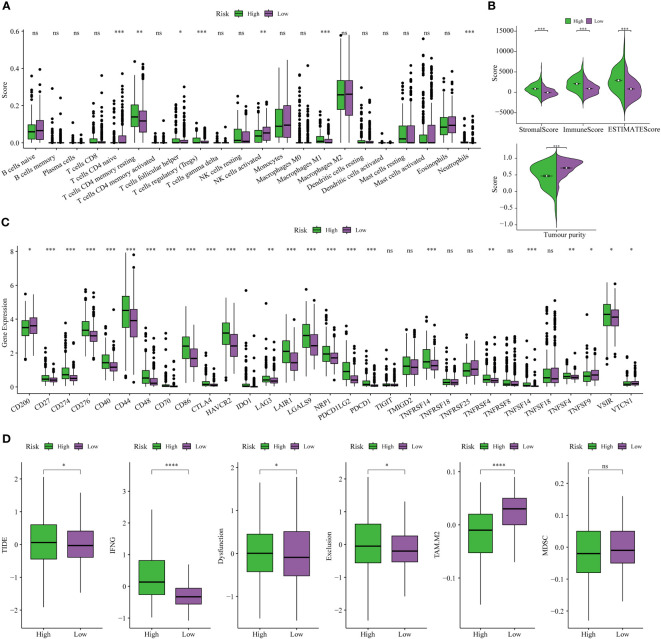
Analysis of immune traits in the training cohort. **(A)** Distribution patterns of 22 tumor-infiltrating immune cells in the training set. **(B)** Analysis of components in the tumor microenvironment (TME) between the two risk subgroups. **(C)** Expression patterns of immune checkpoint genes in the training cohort. **(D)** The Tumor Immune Dysfunction and Exclusion (TIDE) analysis between high-risk and low-risk subgroups of LGG patients in the training cohort. *P< 0.05, **P< 0.01, ***P< 0.001.

The CIBERSORT technique assessed the level of immune cell penetration in each sample, providing insights into the arrangement and interrelation of the relative ratios of 22 immune cells that infiltrated tumors within the TCGA-LGG group. **Figure 8A** provides an overview of the landscape depicting the infiltration patterns of 22 distinct immune cell types. In particular, patients classified in the high-risk group demonstrated elevated levels of regulatory T cells (T-cell regulatory cells and T-cell follicular helper cells) and quiescent immune cells (resting CD4+memory T cells and resting NK cells). Conversely, the proportion of T-cell CD4+ primary cells and activated natural killer cells was notably lower in this group (**Figure 8A**). The TME plays a pivotal role in modulating the immune response against malignant cells. We assessed the composition of the TME between different risk subgroups within the TCGA cohort. Briefly, elevated stromal and immune scores were significantly associated with the high-risk subset compared to the low-risk subgroup. Conversely, tumor purity was markedly lower in the high-risk subgroup (**Figure 8B**). This indicates that high-risk scores are concomitant with increased levels of immune cell infiltration within the TME. Furthermore, the high-risk subgroup exhibited elevated expression levels of most immune checkpoints, while the low-risk subgroup displayed the opposite pattern, as depicted in **Figure 8C**. These data implied that the upregulation of immunosuppressive cells, the inactivation of NK cells, and the expression of immune checkpoints may contribute to establishing an immunosuppressive TME in patients classified as high-risk group.

Finally, the TIDE analysis was conducted to assess the variability in immunotherapeutic responsiveness among patients stratified by distinct risk profiles. Based on the study findings, it was observed that patients categorized in the low-risk group displayed a more favorable response to immunotherapy. This observation can be attributed to their comparatively lower TIDE, Dysfunction, and Exclusion scores, as depicted in **Figure 8D**.

### 3.7 The disulfidptosis-based risk score assesses anti-tumor immune activity

**Figure 9:**
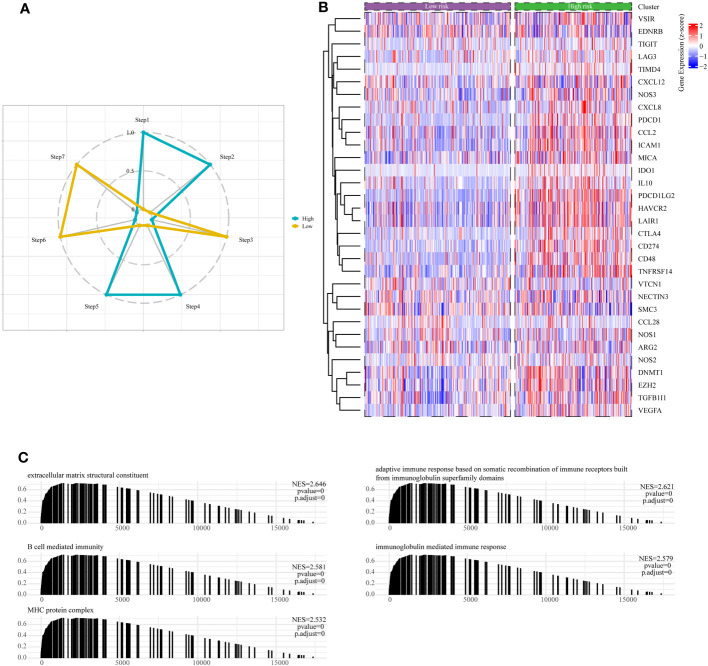
Assessment of anti-cancer immune activity between risk subgroups. **(A)** Differential analysis of anti-tumor immune activity in the seven-step tumor-immunity cycle between high and low-risk subgroups. **(B)** Heatmap showing the expression patterns of genes involved in the negative regulation of immune processes between high- and low-risk subgroups. **(C)** The Gene Set Enrichment Analysis (GSEA) reveals the underlying biological processes associated with the high- and low-risk subgroups.

The cancer immunotherapy field is guided by the conceptual framework of the 7-step cancer-immunity cycle, which is currently driving modern research (21). Using TIP, a web tool for evaluating tumor immunophenotypes (26), we examined the immune response against cancer in various risk subcategories of LGG patients by analyzing the seven-step cancer-immunity cycle. In patients belonging to the high-risk subgroup, there was notable heightened activity observed at steps 1 (pertaining to the release of tumor antigens), 2 (related to cancer antigen presentation), 4 (involving T-cell migration to the tumor site), and 5 (linked to immune cell penetration into the TME). Conversely, steps 3 (characterizing initiation and activation processes of immune cells), 6 (involving T-cell recognition of tumor cells), and 7 (concerning the killing of tumor cell) exhibited distinctive inhibitory characteristics (**Figure 9A**). Contrarily, patients categorized within the low-risk subgroup displayed augmented activity levels at steps 3, 6, and 7, while showing discernible inhibition at steps 1, 2, 4, and 5, as depicted in **Figure 9A**. These results suggested ameliorating the immunosuppressive conditions within the high-risk subgroup of patients, as well as enhancing immune cell infiltration among individuals within the low-risk subgroup, could potentially be conducive to achieving favorable clinical outcomes in patients with LGG.

Moreover, it is essential to note that the majority of genes implicated in the negative regulation of immune processes exhibited upregulation within the high-risk subgroup of LGG patients, as visually represented in **Figure 9B**. Finally, we conducted an in-depth examination of potential pathways linked to risk subgroups through GSEA enrichment analysis. The outcomes unveiled a significant enrichment of negative regulation of immune pathways within the high-risk subgroups, encompassing pathways such as B-cell-mediated humoral immunity, cellular immunity, adaptive immune response, and immunoglobulin-mediated immune response (**Figure 9C**).

### 3.8 Somatic mutation landscapes in high- and low-risk subsets of LGG individuals

**Figure 10:**
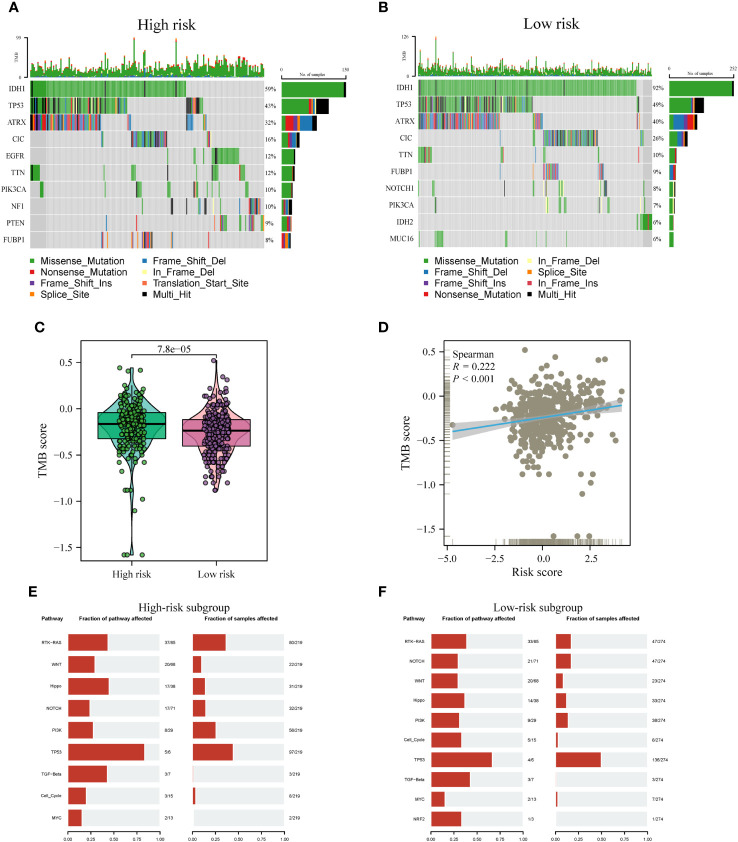
Comparison of somatic mutations between risk subtypes. **(A, B)** Waterfall plots visualizing the top 10 most frequently mutated genes in the high-risk **(A)** and low-risk **(B)** subgroups. **(C)** Divergence in tumor mutational burden (TMB) levels across the high- and low-risk subgroups. **(D)** Correlativity betwixt TMB and risk scores. **(E, F)** Mutant oftenness of nine common oncogenic pathways between high- **(E)** and low-risk subgroups **(F)**.

Distinct patterns of somatic mutations were identified between the high- and low-risk subgroups. Despite IDH mutations being the most prevalent, the relative frequency of IDH mutations exhibited variations across subtypes. Among patients within the low-risk subgroup, IDH1 mutations were notably more frequent, constituting 92% of the total mutations. In contrast, patients within the high-risk subgroup displayed a lower proportion, with IDH mutations accounting for only 59% of the total mutations, as illustrated in **Figures 10A, B**. Furthermore, it is noteworthy that the highest incidence of TP53 mutations was observed within the low-risk subgroup, accounting for 49%, followed closely by the high-risk subgroup, where TP53 mutations constituted 43% of the mutations. Tumor Mutation Burden (TMB) levels exhibited significant disparities between the two risk subgroups, with a noteworthy positive correlation observed between risk scores and TMB values, as depicted in **Figures 10C, D**.

Subsequent investigations involved the assessment of mutation frequencies within some major oncogenic pathways among the two risk subgroups. The outcomes revealed that the RTK-RAS, Hippo, and TP53 pathways were predominantly identified in the high-risk subgroup, as illustrated in **Figure 10E**. Conversely, the low-risk subgroup showed a predominant detection of the Notch and Cell Cycle pathways, as shown in **Figure 10F**, whereas the other pathways did not display notable disparities between the two subgroups.

### 3.9 Validation of DAGs expression patterns in the risk model through scRNA-seq analysis

**Figure 11:**
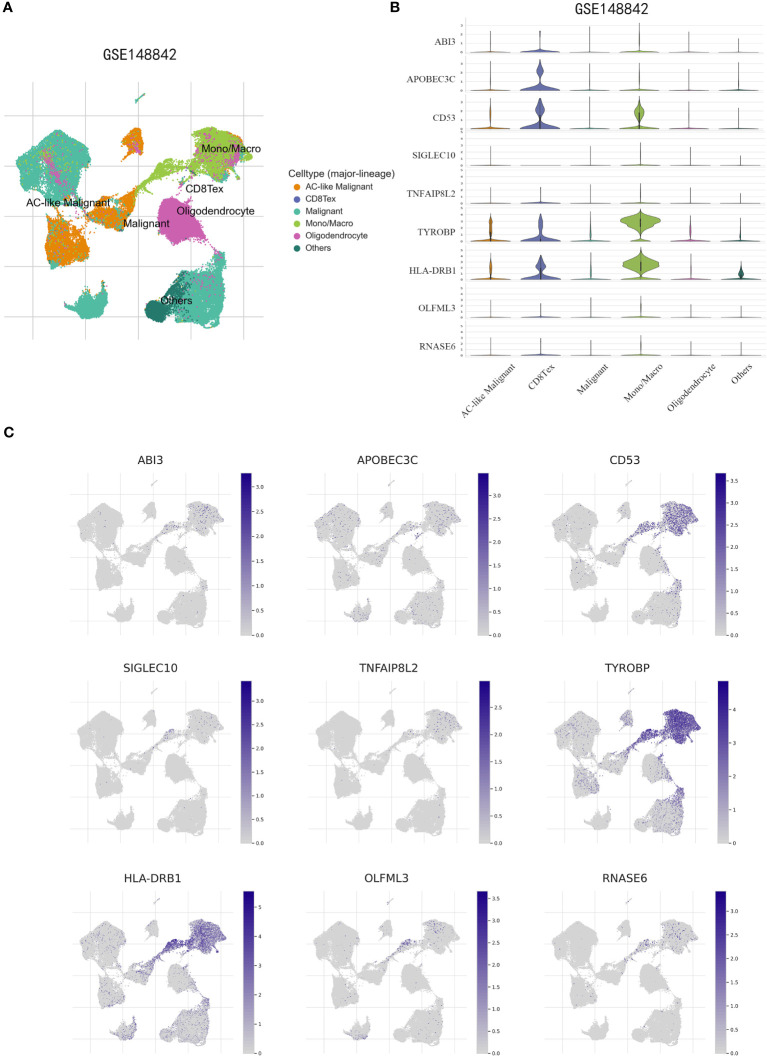
scRNA-Seq revealing the expression patterns of disulfidptosis-associated genes (DAGs) at the single-cell level. **(A)** The UMAP plot annotates the cells into six disparate cell types. **(B, C)** Violin plots **(B)** and UMPA **(C)** plots show dissimilar expression patterns of DAGs within the prognostic signature.

To obtain further validation on the particular cell types that express the genes in the risk model within the TME, scRNA-seq analysis was performed using the TISCH online tool on the GSE148842 dataset. A total of six distinct cell clusters were identified through UMAP analysis, which included the following categories: AC-like Malignant cells, CD8Tex cells, Malignant cells, Monocytes/macrophages, Oligodendrocyte, and other types of cells (**Figure 11A**). The findings revealed distinct patterns of gene expression within the identified cell clusters. Specifically, ABI3 exhibited predominant expression in malignant cells and monocytes/macrophages, while APOBEC3C was primarily expressed in CD8Tex cells. CD53, TYROBP, and HLA-DRB1 showed predominant expression in monocytes/macrophages, CD8Tex cells, and malignant cells. In contrast, SIGLEC10, TNFAIP8L2, OLFML3, and RNASE6 displayed low expression levels in non-tumorigenic and tumorigenic cells (**Figures 11B, C**).

### 3.10 Expression levels of ABI3 in glioma tissue samples and its silencing efficacy in cultured glioma cell lines

**Figure 12:**
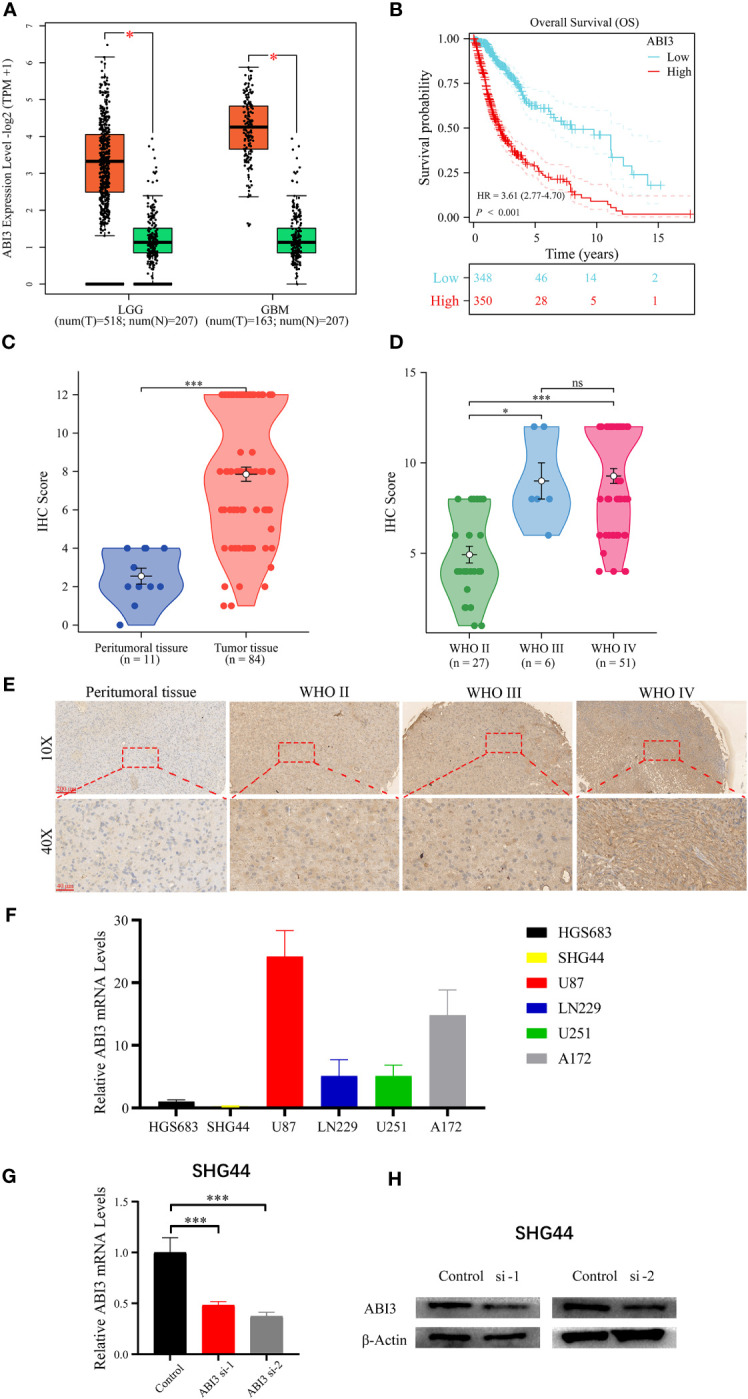
Analyzing and validating ABI3 expression. **(A)** Significantly higher mRNA levels of ABI3 in lower-grade glioma (LGG) and glioblastoma (GBM) tissues compared to normal tissues. **(B)** Survival analysis indicates that glioma patients with high ABI3 expression have a significantly worse prognosis. **(C)** Quantitative immunohistochemical (IHC) scores revealed significantly reduced ABI3 protein expression in paraneoplastic tissues compared to tumoral tissues. **(D)** Quantifying ABI3 protein expression across varied grades of diffuse glioma specimen via IHC. **(E)** Representative IHC images of ABI3 in diverse grades of glioma and peritumoral tissues. **(F)** The mRNA levels of ABI3 in six glioma cell lines (HGS683, SHG44, U251, LN229, U87 and A172). **(G, H)** Assessment of silencing efficiency of two ABI3-specific siRNAs via RT-qPCR **(G)** and Western blot **(H)** in SHG44 cell line. *P< 0.05, ***P< 0.001, ns indicates no significant difference.

Utilizing the GEPIA2 online platform (27), we assessed the expression profile of ABI3 in glioma and normal tissue samples. Significantly, ABI3 expression in glioma tissues was notably higher when compared to their normal counterparts (**Figure 12A**). Furthermore, analysis of the survival curve indicated that increased expression was linked to an adverse prognosis in patients with glioma (**Figure 12B**). In order to validate ABI3 expression levels in glioma tissues, we conducted immunohistochemistry experiments on tissue microarrays made from glioma specimens collected from Xiangya Hospital (containing 11 paraneoplastic tissues and 84 diffuse glioma samples). The experimental data demonstrated a significant diminution in ABI3 protein expression in paraneoplastic tissues relative to tumor tissues (**Figure 12C**). Moreover, there was a significant increase in protein expression of ABI3 in WHO grade III (*P<* 0.05) and grade IV gliomas (*P<* 0.001) compared to WHO grade II gliomas (**Figure 12D**). Nevertheless, no significant statistical difference in protein expression levels was observed between gliomas of WHO grade III and grade IV. Immunohistochemical data pertaining to ABI3 expression are presented in **Figure 12E**.

To elucidate the biological role of ABI3 in glioma, we initially assessed the mRNA expression levels of ABI3 across six glioma cell lines, namely HGS683, SHG44, U251, LN229, U87 and A172 (**Figure 12F**). Among them, the GBM cell lines exhibited relatively higher ABI3 mRNA levels than the LGG cell lines. We chose the LGG cell line SHG44 and the GBM cell line U251 for subsequent cell experiments. We validated the effective downregulation of ABI3 expression in SHG44 using siRNA-1 and siRNA-2 through qPCR and Western blot analysis (**Figures 12G, H**).

### 3.11 ABI3 is implicated in the migratory and invasive behaviors of glioma cells but not in cellular proliferation

**Figure 13:**
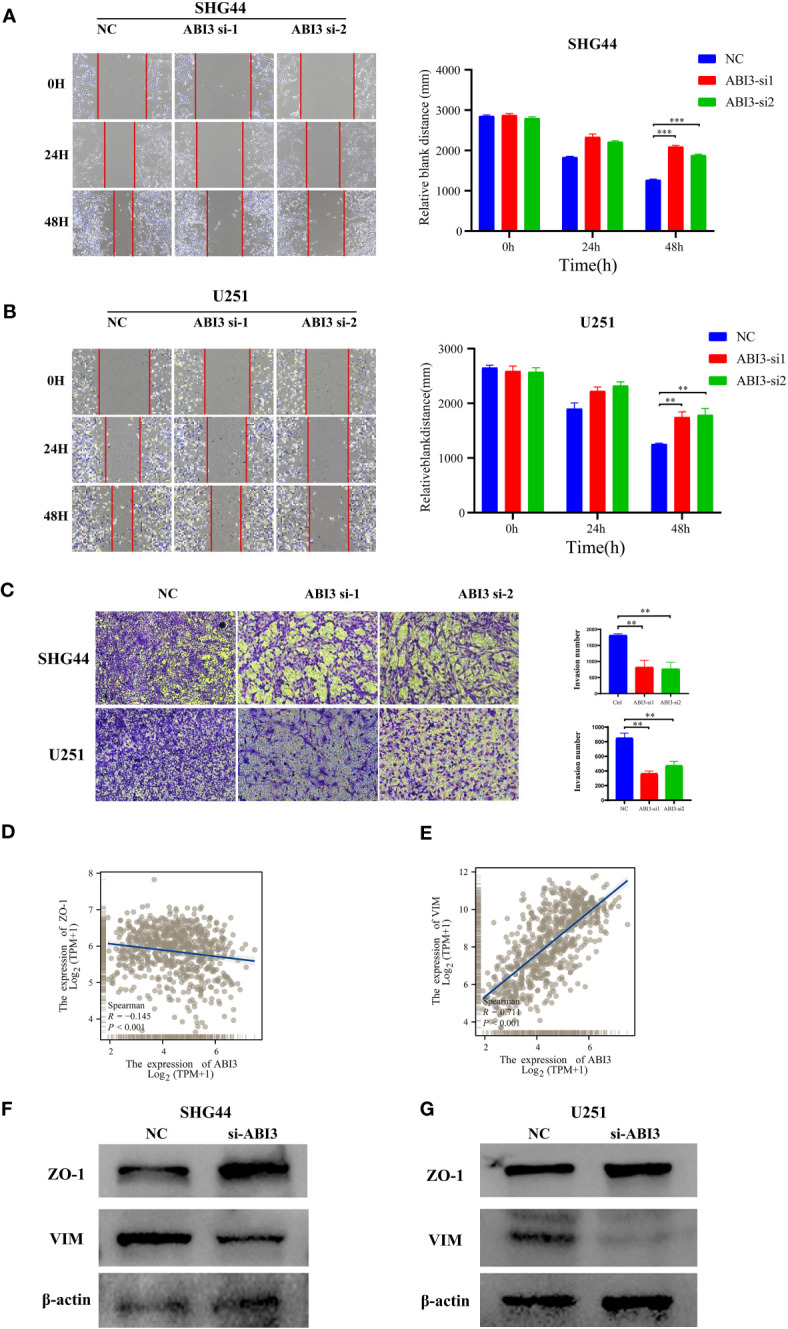
ABI3 knockdown inhibiting the mobility of glioma cell lines. **(A, B)** The wound healing assay showed that ABI3 knockdown significantly inhibited the migration of SHG44 **(A)** and U251 cells **(B)**. **(C)** The downregulation of ABI3 significantly reduced the invasion capabilities of SHG44 and U251 cells. **(D, E)** Correlation analysis betwixt ABI3 and Epithelial-mesenchymal transition (EMT) markers such as ZO-1 **(D)** and VIM **(E)** protein expression via Spearman’s method. **(F, G)** Western blot demonstrating changes in the expression of EMT markers (ZO-1 and VIM protein) in the SHG44 and U251 cells after knockdown of the ABI3 gene with siRNA. **P< 0.01, ***P< 0.001.

Further loss-of-function experiments were conducted on SHG44 and U251 cells. LGG cell line SHG44 and the GBM cell line U251 were segregated into a negative control group (NC) and an ABI3-silenced group (wherein ABI3 expression was suppressed in both SHG44 and U251 cell lines). Subsequent scratch assays and Transwell matrigel invasion assays revealed that ABI3 downregulation notably attenuated the migratory and invasive capabilities of SHG44 and U251 glioma cells (**Figures 13A–C**). Subsequently, we performed a correlation analysis between ABI3 expression and molecular markers indicative of epithelial-mesenchymal transition using the TCGA database. The analysis disclosed a significant negative association between ABI3 expression and the epithelial marker ZO-1 (**Figure 13D**), as well as a pronounced positive correlation with the mesenchymal marker vimentin (**Figure 13E**). To corroborate these observations, we quantified the expression levels of ZO-1 and vimentin via Western blot analysis. The experimental data revealed an upregulation of ZO-1 expression and a downregulation of vimentin expression following ABI3 interference in both SHG44 and U251 cell lines (**Figures 13F, G**). These findings suggest that overexpression of ABI3 may serve as a contributory factor in the facilitation of glioma cell invasion and metastasis.

Regarding cellular proliferation, CCK8 assay results indicated a moderate reduction in the proliferative capacity of SHG44 cells (**Supplementary Figure 1A**) following the interference with ABI3 expression. However, the proliferation of the U251 cell line (**Supplementary Figure 1B**) remained unaffected. Colony formation assay results demonstrated that interference with ABI3 expression in SHG44 and U251 cells did not yield significant differences in the number and size of cell colonies compared to the control group (**Supplementary Figures 2C, D**). These findings suggested that the knockdown of the ABI3 gene exerted a minimal impact on the proliferation of glioma cells.

### 3.12 Suppression of ABI3 expression augments disulfidptosis in glioma cells

**Figure 14:**
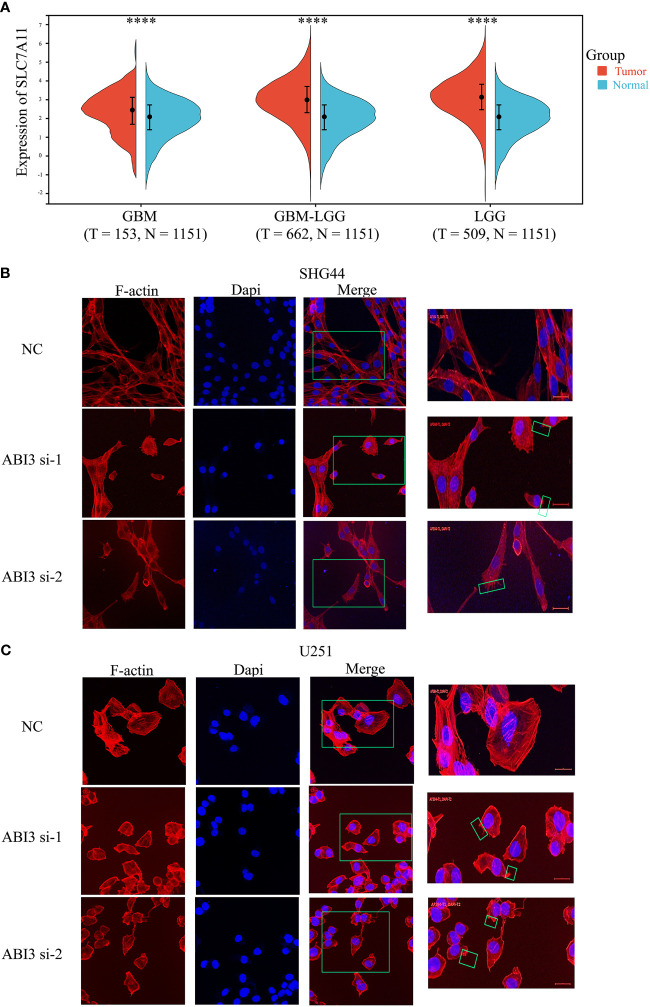
ABI3 involved in glioma cell disulfidptosis. **(A)** Detecting the expression of the SLC7A11 gene in glioma tissues via Sangerbox 3.0 Website. **(B, C)** Confocal images showing cytoskeletal contraction and lamellipodia formation labeled by Phalloidin after silencing of ABI3 in SHG44 **(B)** and U251 cells **(C)**. Blue: cell nucleus labeled by DAPI; Red: F-actin labeled by Phalloidin; Green box: the phenomenon of cytoskeletal contraction and lamellipodia formation. ****P< 0.0001.

Using the expression profile data from TCGA and GTEx, we analyzed the mRNA levels of SLC7A11 in patients with glioma. The findings indicated a significant upregulation of SLC7A11 in the tumor samples, as depicted in **Figure 14A**. Moreover, studies have shown that increased SLC7A11 levels contributed to the malignant progression and unfavorable prognosis of glioma (8, 28). As a result, we substituted the DMEM medium with the low-glucose 1640 medium for both the control group (NC) and the experimental group. Following 48 hours, we proceeded to stain the cytoskeleton using phalloidin. During the examination under confocal microscopy, it was observed that the suppression of ABI3 gene expression led to the contraction of the cytoskeleton and the formation of lamellipodia in SHG44 (**Figure 14B**) and U251 cells (**Figure 14C**). The findings suggested that ABI3 may act as a potential target in disulfidptosis.

## 4 Discussion

Gliomas, being the most prevalent malignant brain tumors, exert a substantial detrimental impact on patients’ quality of life and impose a significant economic burden on society (29). LGG patients currently have limited advantages from conventional treatments. Furthermore, individuals suffering from LGG still face significant problems due to the ongoing difficulties of postoperative recurrence and drug resistance. The main contributing factor to the poor prognosis and treatment results seen in LGG is thought to be the highly diverse and complex nature of the TME (30).

Disulfidptosis is a recently discovered type of programmed cellular demise. The initiation of abnormal cross-linking among actin cytoskeletal proteins occurs due to the excessive accumulation of disulfides, particularly cysteine, throughout this process. This event leads to the contraction of the cytoskeleton, which disrupts its structural arrangement and ultimately leads to the collapse of the actin network, resulting in the subsequent death of the cell. As a tactic to avoid disulfidptosis, tumor cells accelerate the transformation of consumed cystine into cysteine (31). Several research studies have presented proof of the possible benefit in focusing on proteins associated with disulfidptosis for therapeutic interventions in tumor treatment (7, 9). Moreover, specific research has suggested potential uses of disulfides in certain cancer treatments. For instance, anti-tumor medications like cisplatin and paclitaxel attain their curative advantages by engaging with intracellular disulfides (32, 33). Furthermore, numerous studies have presented convincing proof that SLC7A11 plays a significant role in promoting resistance to ferroptosis and has a vital regulatory function in relation to tumors and various disease conditions (34, 35).

Compared with other existing predictive models for glioma, such as the studies of Wan RJ et al. (32) and Zeng Z et al. (33), our model is specifically designed around the emerging concept of disulfidptosis. This focus allows for a deeper exploration of novel pathological mechanisms specific to LGG, potentially offering insights that are not covered by models focusing on more general or well-established pathways. Regarding the data analysis methods in uncovering the functional role of DAGs in LGG, this study utilized a combination of WGCNA and ssGSEA for investigation. By utilizing a combination of Cox regression, Lasso regression, and SVM-RFE algorithms, a total of nine Directed Acyclic Graphs (DAGs) were systematically identified. Afterward, these nine chosen genes created a new marker linked to disulfideptosis for LGG. The predictive algorithm calculates personalized risk scores for patients and categorizes them into two risk-defined subcategories—high and low—by utilizing the median risk value as a dividing point. The group at high risk shows significantly worse prognostic outcomes than those at low risk. In order to verify the accuracy of the model, ROC curves were performed on the TCGA training set as well as the two CGGA validation sets. At the 3, 5, and 7-year milestones, the AUC values exceeded 0.72. Notably, the highest AUC value detected was 0.87, observed at the three-year point. Like the well-known clinicopathologic features of gliomas including age and 1p/19q co-deletion, our predictive model has become a separate prognostic factor for patients diagnosed with LGG. Following this, prognostic factors that are independent and relevant to patients with LGG were identified in order to create a nomogram, which would improve the evaluation of prognostic outcomes specific to each patient. To validate the predictive precision of the developed nomogram, calibration curves, and decision curve analyses were utilized. This enhanced predictive capability is crucial for personalized patient management and treatment planning.

In addition, by implementing prognostic models on a server accessible via the internet, we make it easier for both researchers and clinicians to obtain estimated probabilities of OS. Gliomas’ onset and advancement are significantly impacted by the TME (24). The TME of glioma consists of an intricate cellular environment encompassing immune cells, endothelial cells, neoplastic cells, and various immune-related substances released by these cellular elements. The cellular composition of the immune system in the glioma TME includes macrophages, microglia, dendritic cells, neutrophils, T lymphocytes, and NK cells. The neoplastic cells interact with these cellular components, and the cellular components have a regulatory function in modifying the immune response within the TME (36). An analysis of the TME was performed on patient subgroups categorized as high- and low-risk. The findings indicated that patients in the high-risk subset exhibited higher immune scores, enhanced stromal scores, elevated ESTIMATE scores, and lower tumor purity. By employing the CIBERSORT algorithm for examination, we noticed a heightened abundance of quiescent memory T cells and regulatory T cells in the subgroup of high-risk patients. Tregs act as suppressive cells that not only trigger cell death in effector T cells but also hinder their signaling pathways through the release of inhibitory cytokines (37, 38). Cancer therapeutics can effectively target immune checkpoints, and inhibitors that hinder crucial checkpoint molecules have shown significant effectiveness against neoplastic diseases (39). The current investigation examined a set of 31 genes associated with immune checkpoints, indicating that most of these checkpoints were elevated in the subgroup of patients at high risk. The evaluation of TIDE scores suggests a higher probability of immune evasion in the high-risk patient subset who undergo immunotherapy (40). Moreover, specific genes that suppress the immune system were found to be overexpressed in the subgroup of patients at high risk. These findings suggest that the increased presence of cells that suppress the immune system, inactive immune cells, and immune checkpoints may create an environment that suppresses the immune response in the subgroup of high-risk patients, thus negatively affecting the effectiveness of immunotherapy in this group. Elevated levels of TMB in gliomas serve as an independent prognostic indicator, suggesting a less favorable survival outcome (41). Our research revealed a favorable correlation between TMB and risk assessments in line with previous investigations. Furthermore, TMB has demonstrated the ability to predict the effectiveness of immune checkpoint inhibitors in treating advanced cancers (42). Moreover, we identified potential small molecule therapeutics targeting LGG, including Dasatinib, Gemcitabine, PD0325901, and Selumetinib. Further experimental verification is necessary to determine the effectiveness of these substances in treating LGG.

Among the nine genes employed for signature construction, ABI3 exhibited the most elevated risk factor and was highly expressed in glioma tissues. Based on our empirical evidence, we observed a successful reduction in the expression of the ABI3 gene, which led to a significant inhibition of migration and invasion abilities in glioma cells. The ectopic localization of the WAVE complex (43), a crucial component in the formation of the WAVE Regulatory Complex (WRC) (44), is caused by the expression of ABI3. The WRC-mediated actin cytoskeleton assembly, forming lamellipodia, provides a stress target for disulfides (44–46). Our experimental results showed that when the ABI3 gene was interfered with, glioma cells exposed to glucose deprivation exhibited significant changes in their cytoskeleton, which were observed through immunofluorescence staining of F-Actin. The alterations comprised notable cytoskeleton contraction, separation from the cellular membrane, and the development of protrusions resembling lamellipodia. Disulfide-induced cell death may be modulated by ABI3 and could serve as a therapeutic target. Nonetheless, further investigation is required to uncover the precise mechanisms that occur before and after this point. Furthermore, it is vital to acknowledge the limitations of this study. The utilization of glioma data obtained from the TCGA and CGGA public databases in our research does not adhere to the recent classification criteria established by WHO in 2021 (47). Moreover, we did not investigate the correlation between disulfidptosis and various molecular subtypes in the latest typing of gliomas. In future investigations, it is imperative to incorporate the latest WHO classification of gliomas.

## 5 Conclusions

We constructed an LGG prognostic signature centered on disulfidptosis, demonstrating robust predictive capabilities for factors including patient prognosis and immunotherapeutic response. This signature holds promise for future LGG treatment applications, facilitating the early identification of high-risk patients and screening candidates suitable for immunotherapy to enhance survival outcomes. Additionally, we identified ABI3 as a critical component of the signature. Characterized by elevated expression in glioma, ABI3 is implicated in the migration, invasion, and disulfidptosis of glioma cells. These insights have, to a degree, informed the development of targeted therapeutic strategies for LGG.

## Data availability statement

The original contributions presented in the study are included in the article/**Supplementary Material**. Further inquiries can be directed to the corresponding authors.

## Ethics statement

The study involving human subjects was approved by the Ethics Committee of Xiangya Hospital. These studies are carried out in accordance with local legislation and institutional requirements. The human samples used in this study came from a part of our previous study, which was ethically endorsed. In accordance with national legislation and institutional requirements, participants or their legal guardians / close relatives do not require written informed consent to participate.

## Author contributions

YaZ: Visualization, Writing – original draft. YC: Visualization, Writing – original draft, Conceptualization, Writing – review & editing. WL: Writing – review & editing. LW: Writing – review & editing. YK: Resources, Visualization, Writing – review & editing. YiZ: Resources, Writing – review & editing. QC: Resources, Writing – review & editing. ZC: Resources, Visualization, Writing – review & editing. HH: Resources, Writing – review & editing. WZ: Resources, Writing – review & editing. XJ: Conceptualization, Writing – review & editing. BW: Writing – review & editing. CR: Writing – review & editing.
