# Supplementary figures and images for: Leveraging a gene signature associated with disulfidptosis identified by machine learning to forecast clinical outcomes, immunological heterogeneities, and potential therapeutic targets within lower-grade glioma
Source: Front Immunol. 2023 Dec 15;14:1294459. doi: 10.3389/fimmu.2023.1294459 (PMC10757341; doi:10.3389/fimmu.2023.1294459)

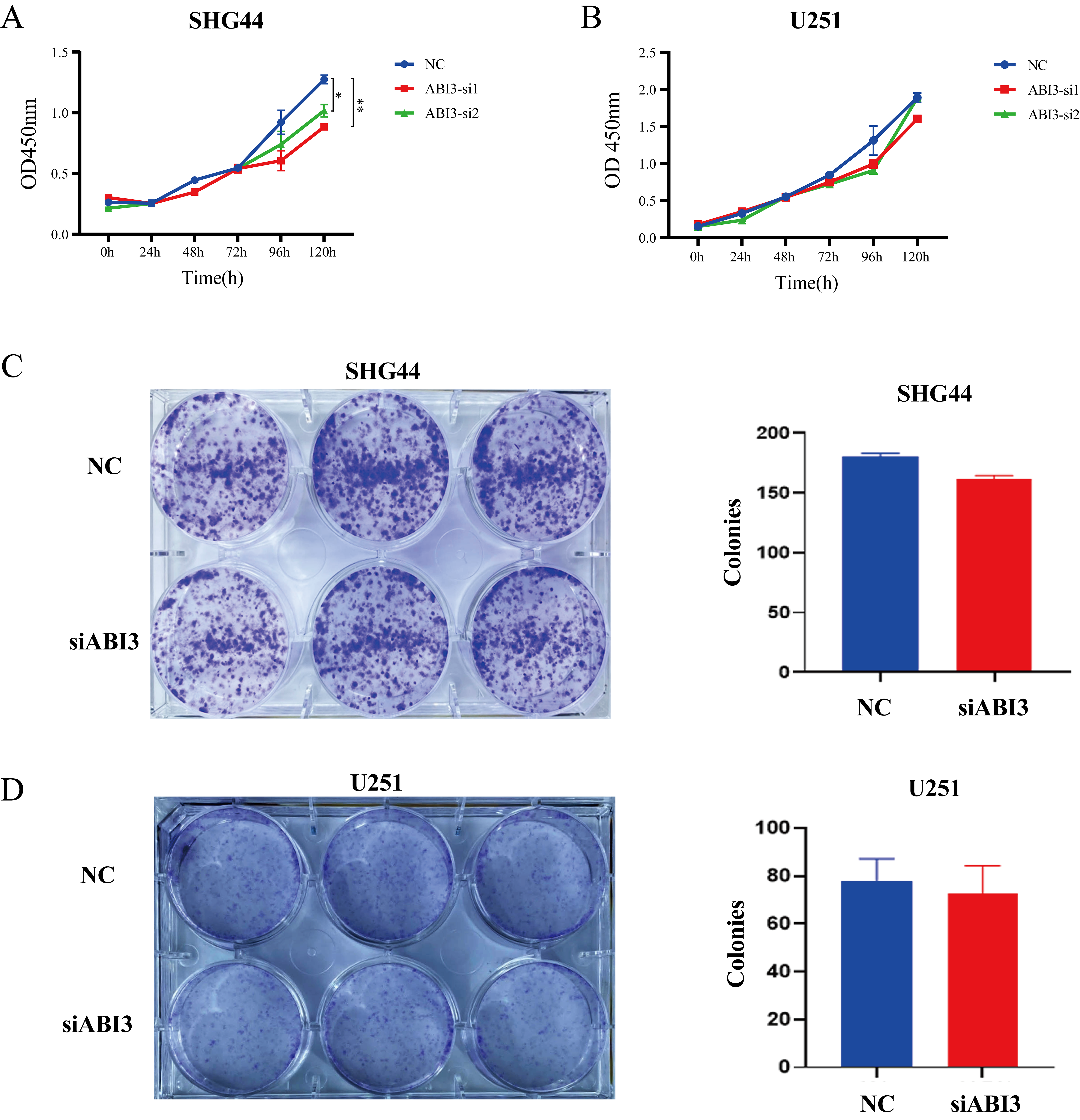

Supplement: Supplementary file 1 [file Image_1.tif]
